# ASM Launches *mSphere*

**DOI:** 10.1128/mSphere.00006-15

**Published:** 2015-11-04

**Authors:** Michael J. Imperiale

**Affiliations:** Department of Microbiology and Immunology, University of Michigan Medical School, Ann Arbor, Michigan, USA

## EDITORIAL

We live in an exceptionally dynamic and exciting time for the life sciences, in which our ability to acquire and disseminate knowledge is rapid, global, and ever-changing. Technological advances have altered the way in which most life sciences laboratories operate. When I was a postdoc, substantial effort was needed to master basic molecular biology techniques that were complicated at the time but which are now available in kits. Today, one must be conversant in next-generation sequencing, total internal-reflection fluorescence microscopy, and bioinformatics. Research is growing more collaborative and interdisciplinary. ASM leadership recognized this in developing the concept for *mSphere*: an open-access panmicrobial sciences journal that will be nimble and responsive to authors, reviewers, and readers.

Our primary goals in designing the operation of the journal were to ensure that we publish high-quality science and that we make the author experience as painless as possible.

The quality will come from a robust editorial and peer review mechanism similar to what we have come to expect from an ASM journal.

We will enhance the author experience by empowering our editors to make rapid decisions. It is our goal to rely on two reviews of each manuscript and to obtain those reviews quickly. Therefore, if you are asked to review a manuscript for *mSphere* and you are busy, please turn us down! It is much preferable for you to say no than to agree to review the manuscript and then take a long time to do so. When the reviews come in, the editor will make a decision. If authors are asked to revise their manuscript, we will not ask for extensive additional experimentation and I will expect our editors to make a decision on the revised manuscript without sending it out for rereview. While there will, of course, be exceptions in some situations, the vast majority of decisions will be guided by these principles. Accepted manuscripts will be posted continuously; there will not be regularly spaced issues, except for indexing purposes.

In addition to publishing primary research results, *mSphere* will provide a broader service to the scientific community. We will feature types of articles that will be useful and of interest to working scientists, as well as to other stakeholders in the microbial science research enterprise, including policy makers, ethicists, and even the public at large.

Those of you who are familiar with the great University of Michigan football head coach Bo Schembechler know that his mantra was, “The team, the team, the team.” We have assembled a team of energetic, insightful, and thoughtful senior editors and editors committed to the goals I have described, and the staff at the ASM Journals Department are equally enthusiastic and dedicated to these goals. We are here to ensure that *mSphere* will be your journal of choice for both presenting your own and reading about others’ cutting-edge scientific results. We are also committed to moving forward with a watchful eye on the dynamic environment in which research functions, and we will evolve to continually meet the needs of the community.

I must thank two people in particular whose vision resulted in the creation of *mSphere* and who have helped me and the rest of the team shape the practices described above: Tom Shenk, Chair of the ASM Journals Board, and Barbara Goldman, ASM Director of Journals. I am grateful for the trust they have placed in me to lead this amazing team.

The senior editors and I look forward to seeing the great science and ideas that you will publish with us!

